# Identification of TNIP1 Polymorphisms by High Resolution Melting Analysis with Unlabelled Probe: Association with Systemic Lupus Erythematosus

**DOI:** 10.1155/2012/265823

**Published:** 2012-07-19

**Authors:** Jie Zhang, Yuewen Chen, Yong Shao, Qi Wu, Ming Guan, Wei Zhang, Jun Wan, Bo Yu

**Affiliations:** ^1^Shenzhen Key Lab for Translational Medicine of Dermatology, Shenzhen PKU-HKUST Medical Center, No. 1120 Lian-Hua Road, Futian District, Shenzhen, Guangdong 518036, China; ^2^Department of Dermatology, Shenzhen Hospital, Peking University, Shenzhen, Guangdong 518036, China; ^3^Biomedical Research Institute, Shenzhen PKU-HKUST Medical Center, No. 1120 Lianhua Road, Futian District, Shenzhen, Guangdong 518036, China; ^4^JNU-HKUST Joint Laboratory, Jinan University, Guangdong 510632, China; ^5^Department of Clinical Laboratory, Shanghai Worldwide Medical Center, Huashan 200040, China; ^6^Division of Life Science, The Hong Kong University of Science and Technology, Hong Kong

## Abstract

*Background*. TNF**α**-induced protein 3 (TNFAIP3) interacting with protein 1 (TNIP1) acts as a negative regulator of NF-**κ**B and plays an important role in maintaining the homeostasis of immune system. A recent genome-wide association study (GWAS) showed that the polymorphism of TNIP1 was associated with the disease risk of SLE in Caucasian. In this study, we investigated whether the association of TNIP1 with SLE was replicated in Chinese population. *Methods*. The association of TNIP1 SNP rs7708392 (G/C) was determined by high resolution melting (HRM) analysis with unlabeled probe in 285 SLE patients and 336 healthy controls. *Results*. A new SNP rs79937737 located on 5 bp upstream of rs7708392 was discovered during the HRM analysis. No association of rs7708392 or rs79937737 with the disease risk of SLE was found. Furthermore, rs7708392 and rs79937737 were in weak linkage disequilibrium (LD). Hypotypes analysis of the two SNPs also showed no association with SLE in Chinese population. *Conclusions*. High resolution melting analysis with unlabeled probes proves to be a powerful and efficient genotyping method for identifying and screening SNPs. No association of rs7708392 or rs79937737 with the disease risk of SLE was observed in Chinese population.

## 1. Introduction

Systemic lupus erythematosus (SLE) is a chronic inflammatory autoimmune disease influenced by both genetic and environmental factors [1, 2]. Recently, the genetic background of this complex disease was robustly revealed by a series of genome-wide association studies (GWAS) [3]. Several SLE susceptive genes including MHC, BLK, ITGAM, STAT4, IRF5, BANK1, and ETS1 were identified although the function of these candidate genes in the pathological development of SLE was still largely unknown [4–10]. Among these candidate genes, the polymorphisms on TNF*α* induced protein 3 (TNFAIP3 or A20) interacting protein 1 (TNIP1) have been found to associate with the disease risk of several autoimmune diseases including psoriasis and SLE [8, 9, 11–15]. TNIP1, also known as A20 binding inhibitors of NF-*κ*B (ABIN1), can interact with TNFAIP3 and I*κ*B kinase *γ*/NF-*κ*B essential modulator (IKK*γ*/NEMO) and acts as a negative regulator of NF-*κ*B signal pathway [16]. Additionally, TNIP1 was also involved in inhibiting the processing of the p105, a precursor of NF-*κ*B [17].

It has been reported that SNP rs7708392 (C/G) on 5q33.1 that resides within an intron of TNIP1 is associated with the disease risk of SLE in the Caucasian population [9]. However, whether this association is also presented in Chinese Han population remains unknown. In this study, we examined the polymorphism of SNP rs7708392 (C/G) in 285 patients and 336 normal controls in the Chinese population using high-resolution melting analysis with unlabelled probe.

## 2. Methods

### 2.1. Study Populations

A total of 285 patients (26 males and 259 females; median age 29 years, range 12–55) who fulfilled the American College of Rheumatology criteria for SLE [18] and 336 ethnically matched healthy controls (28 males and 308 females; median age 28 years, range 17–46) were recruited from Shenzhen Hospital, Peking University. The control group had neither family history nor symptoms related to SLE. The study was approved by the institutional review board of the Shenzhen Hospital and written informed consent was taken from all patients.

### 2.2. Genotyping

Genomic DNA was isolated from peripheral blood cells by using Innogent genomic DNA extraction kit (Innogent, China) according to the manufactory instructions. Genotyping was assayed by high resolution melting with unlabeled probe as previously described [19]. Briefly, asymmetric PCR reaction was performed in a volume of 20 *μ*L containing 20 ng of genomic DNA, 1 × PCR buffer (Takara, Japan), 200 *μ*M dNTPs, 0.5 U rTaq DNA polymerase (Takara, Japan), 0.05 *μ*M forward primer, 0.5 *μ*M excess reverse primer, and 0.5 *μ*M C3-blocked probe. The PCR reactions were performed in a S1000 Thermal Cycler (Bio-Rad, USA). The conditions included an initial denaturation at 94°C for 2 min, followed by 50 cycles of 94°C for 30 s, 55°C for 30 s and 72°C for 20 s, and a final extension at 72°C for 5 minutes. The 10 *μ*L of PCR products were supplied with 1 *μ*L LCGreen Plus Dye (Idaho Technology, USA) and transferred to a capillary tube on the HR-1 (Idaho Technology, USA). The samples were first denatured at 95°C for 30 s and rapidly cooled to 40°C for 30 s, then melted from 55°C to 90°C with a 0.3°C/s ramp rate. Melting curves were analyzed with LightScanner software (Idaho Technology, USA). All the primers and probes were designed by Lighscaner probe design software (Idaho Technology, USA). The sequences of the primers used in PCR were as follows: forward 5′-TGG TCA ATT CTC CCA ACC GA-3′, reverse 5′-ACT TCA AGG TCA GAC CCT AAA-3′ and three unlabeled C3 block probes used during the genotyping were listed as follows: probe 5′-GCT GAT TCC AGT TAT TGT GAC TAG TCT ACT-3′, probe-1 5′-CGA GGA GAG GCT GAT TCC AAT TAT T-3′, and probe-2 5′-TTA TTG TGA CTA GTC TAC TAA GTT CCA GA-3′. The position of the primers and probe on the genomic DNA sequence are presented in Figure 2(a), respectively.

### 2.3. Statistical Analysis

The SNP was analyzed for an association with the disease by means of comparison of the minor allele frequency (MAF) in patients and controls as well as the constancy of Hardy-Weinberg equilibrium using chi-square test or Fisher's exact test. The magnitude of association was expressed as odds ratio (OR) with a 95% confidence interval (CI). Linkage disequilibrium (LD) and haplotype analysis were carried out by SHEsis software [20]. *P* values less than 0.05 were considered statistically significant.

## 3. Results

### 3.1. Discovery of a New SNP Located on 5 bp Upstream of SNP rs7708392

**Figure 1:**
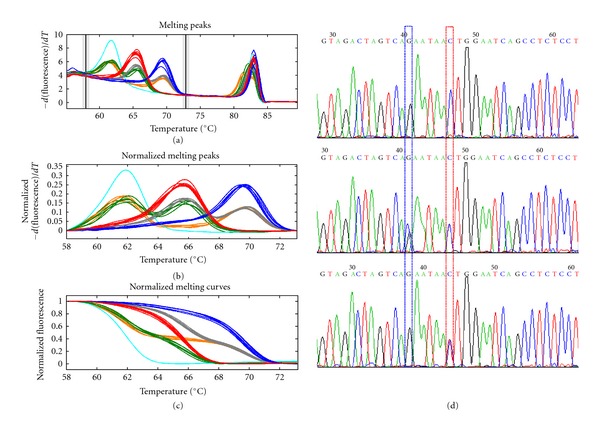
SNP genotyping by HRM with unlabeled probe. (a) Derivative melting curves of unlabeled probe and amplicon for genotyping of SNP rs7708392. (b) Normalized difference curves of unlabeled probe region. (c) Normalized melting curves of unlabeled probe region. Unlike the classical SNP genotyping (wildtype, heterozygote, and homozygote), six types of curves were observed implying a new SNP also presented in probe region. (d) DNA sequencing result of SNP rs7708392. The sequencing was performed by using reverse primer of the PCR amplicon. The blue box indicates the SNP rs7708392 harbors G/C mutation by reverse sequencing. The red box shows that the polymorphism on the new SNP is C/T by reverse sequencing.

High resolution melting analysis with unlabeled probes is a newly developed method for SNP detection with low cost and high efficiency [21, 22]. Single mutation in the genomic DNA sequence could cause mispairing within the unlabeled probe region, which produces a shift of melting peaks. Typically, three kinds of melting curves representing three genotypes (wildtype, heterozygote, and mutant) could be well distinguished. Then, this method was employed to detect the genotypes of SNP rs7708392. Interestingly, more than three melting curves were observed in the melting curves during genotyping (Figure 1), implying that a new unknown SNP might also exist on the probe region. The following DNA sequencing results revealed that a new mutation (G/A) locating on 5 bp upstream of SNP rs7708392 (G/C) was found (Figure 1(d)). This new SNP was submitted to NCBI and assigned an accession number rs79937737 (ss244236678).

**Figure 2:**
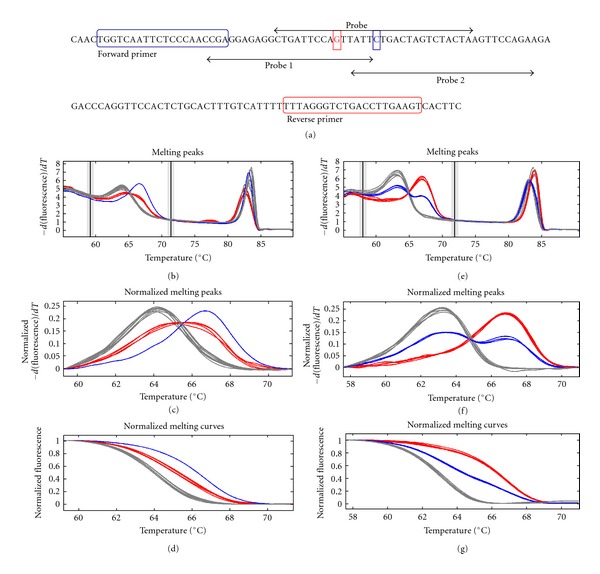
SNP genotyping by HRM with specific probes targeting each SNP, respectively. (a) The location of probes and PCR primers. Probe 1 targets the new-discovered SNP. Probe 2 targets rs7708392. The probe containing both SNPs is also shown (b)–(g). The derivative melting curve, normalized difference curve, and normalized melting curve for each genotyping assay by using probe 1 and probe 2 are shown as indicated.

To discriminate these two SNPs independently, we redesigned two new probes which could specifically match to each SNP, respectively (Figure 2(a)). As presented in Figure 2, both probe 1 and probe 2 could clearly distinguish three genotypes. Then, these probes were used for genotyping all the SLE and normal samples.

### 3.2. No Association of rs7708392 or rs79937737 with the Disease Risk of SLE

Table 1 shows genotype and allele frequencies of SNPs rs7708392 and rs79937737 in SLE patients and healthy controls. Genotype frequencies were in Hardy-Weinberg's equilibrium in the patients and controls. Neither genotype nor allele frequencies of rs7708392 or rs79937737 showed statistically significant differences between SLE patients and controls. Haplotype analysis showed that SNPs rs7708392 and rs79937737 were in weak linkage disequilibrium (LD) (*r* ^2^ = 0.020). Furthermore, all the haplotypes generated from these two SNPs showed no significant association with the disease risk of SLE (Table 2).

## 4. Discussion

In comparison with other traditional SNP screening methods, high melting curve analysis (HRMA) is a powerful and cost-effective method for SNP screening [21, 22]. However, it is occasionally difficult to discriminate the wildtype and homomutant since the melting temperature shifts between these genotypes are almost undetectable (less than 0.4°C). By using a ~30 bp C3-blocked probe to target the SNP of interest, the melting temperature shift could be amplified to 3~4°C which can be much feasible to detect. Moreover, some undiscovered SNPs locating on the probe region could also be observed during the genotyping, which would be helpful for identifying new SNPs. In our case, an unknown SNP existing in the probe region gave rise to a completely new pattern of melting curves. Six types of melting curves were shown up, implying that at least two SNPs were located in the probe region. Even though it was still difficult to deduce which genotype was represented by each melting curve, respectively, the potential advantage of the HRMA with unlabeled probe in identifying new SNPs was well appreciated in our work.

As a negative regulator of NF-*κ*B signal pathway, TNIP1 might play an important role in NF-*κ*B associated innate and adaptive immune response. Until recently, the potential role of TNIP1 during the disease development of SLE has been appreciated since the polymorphism of TNIP1 is associated with the disease risks of SLE in Caucasian population [9]. A recent work reported that rs7708392 was associate with SLE in Japanese population by using a fluorescence probe based TaqMan SNP genotyping assay [15]. In the present work, we found that SNP rs7708392 (G/C) was not relevant to the disease risk of SLE in Chinese population. This discrepancy might cause by the ethnic divergence. Intriguingly, we identified a new SNP (rs79937737) locating on just 5 bp upstream of rs7708392. This new SNP showed low mutation frequency since almost no homo-mutant was observed in our samples. However, these two SNPs were in weak linkage disequilibrium (*r* ^2^ = 0.02). This nonlinkage disequilibrium in such a short distance on the genome indicates that rs79937737 might be a newly developed polymorphism during the evolution of genome. Whether rs79937737 also exists in other populations needs to be carefully investigated in the future.

## Figures and Tables

**Table 1:** Genotype and allele frequencies of TNIP1 SNPs in SLE cases and controls*.

SNP, population	Number of subjects	Genotype frequency, *n* (%)			*P* value	Allele frequency, *n* (%)		*P* value	OR (95% CI)
		Major homozygote	Heterozygote	Minor homozygote		Major allele	Minor allele		
rs7708392									
Genotype or allele		CC	CG	GG		C	G		
Cases	283	179 (63.3)	91 (32.2)	13 (4.6)	0.915	449 (79.3)	117 (20.7)	0.697	1.056 (0.803 ~ 1.389)
Controls	336	207 (61.6)	113 (33.6)	16 (4.8)		527 (78.4)	145 (21.6)		
rs79937737									
Genotype or allele		GG	AG	AA		G	A		
Cases	283	239 (84.4)	43 (15.2)	1 (0.4)	0.500	521 (92.0)	45 (8.0)	0.523	1.148 (0.751 ~ 1.757)
Controls	336	289 (86.0)	47 (14)	0 (0)		625 (93.0)	47 (7.0)		

*SNP: single-nucleotide polymorphism; SLE: systemic lupus erythematosus; OR: odds ratios; 95% CI: 95% confidence interval.

**Table 2:** Haplotype analysis of TNIP1 SNPs in SLE cases and controls*.

Haploptype	Cases, *n* (%)	Controls, *n* (%)	*P* value	OR (95% CI)
AC	44.26 (7.8)	46.84 (7)	0.568	1.132 (0.739 ~ 1.735)
AG	0.74 (0.1)	0.16 (0)	0.590	5.357 (0.272 ~ 105.552)
GC	404.74 (71.5)	480.16 (71.5)	0.983	1.003 (0.783 ~ 1.285)
GG	116.26 (20.5)	144.84 (21.6)	0.664	0.941 (0.715 ~ 1.238)

*SNP: single-nucleotide polymorphism; SLE: systemic lupus erythematosus; OR: odds ratios; 95% CI: 95% confidence interval.
